# Human oocyte area is associated with preimplantation embryo usage and early embryo development: the Rotterdam Periconception Cohort

**DOI:** 10.1007/s10815-023-02803-1

**Published:** 2023-05-02

**Authors:** Rosalieke E. Wiegel, Eleonora Rubini, Melek Rousian, Sam Schoenmakers, Joop S. E. Laven, Sten P. Willemsen, Esther B. Baart, Régine P. M. Steegers-Theunissen

**Affiliations:** 1 Department of Obstetrics and Gynecology, Erasmus MC, University Medical Center, 3015 GD Rotterdam, The Netherlands; 2 Department of Biostatistics, Erasmus MC, University Medical Center, 3015 GD Rotterdam, The Netherlands; 3 Department of Developmental Biology, Erasmus MC, University Medical Center, 3015 GD Rotterdam, The Netherlands

**Keywords:** Oocyte area, In vitro fertilization, Assisted reproduction, Morphological marker, Early life course

## Abstract

**Purpose:**

To investigate the association between oocyte area and fertilization rate, embryo usage, and preimplantation embryo development in order to establish if oocyte area can be a marker for optimal early embryo development.

**Methods:**

From 2017 to 2020, 378 couples with an indication for IVF (*n* = 124) or ICSI (*n* = 254) were included preconceptionally in the Rotterdam Periconception Cohort. Resulting oocytes (*n* = 2810) were fertilized and submitted to time-lapse embryo culture. Oocyte area was measured at the moment of fertilization (t0), pronuclear appearance (tPNa), and fading (tPNf). Fertilization rate, embryo usage and quality, and embryo morphokinetics from 2-cell stage to expanded blastocyst stage (t2-tEB) were used as outcome measures in association with oocyte area. Oocytes were termed “used” if they were fertilized and embryo development resulted in transfer or cryopreservation, and otherwise termed “discarded”. Analyses were adjusted for relevant confounders.

**Results:**

Oocyte area decreased from t0 to tPNf after IVF and ICSI, and oocytes with larger area shrank faster (β − 12.6 µm^2^/h, 95%CI − 14.6; − 10.5, *p* < 0.001). Oocytes that resulted in a used embryo were larger at all time-points and reached tPNf faster than oocytes that fertilized but were discarded (oocyte area at tPNf in used 9864 ± 595 µm^2^ versus discarded 9679 ± 673 µm^2^, *p* < 0.001, tPNf in used 23.6 ± 3.2 h versus discarded 25.6 ± 5.9 h, *p* < 0.001). Larger oocytes had higher odds of being used (oocyte area at tPNf OR_used_ 1.669, 95%CI 1.336; 2.085, *p* < 0.001), were associated with faster embryo development up to the morula stage (e.g., t9 β − 0.131 min, 95%CI − 0.237; − 0.025, *p* = 0.016) and higher ICM quality.

**Conclusion:**

Oocyte area is an informative marker for the preimplantation development of the embryo, as a larger oocyte area is associated with higher quality, faster developing embryos, and higher chance of being used. Identifying determinants associated with oocyte and embryo viability and quality could contribute to improved preconception care and subsequently healthy pregnancies.

**Supplementary Information:**

The online version contains supplementary material available at 10.1007/s10815-023-02803-1.

## Introduction

The earliest stages of embryo development are sensitive to changes in oocyte volume [1]. Volume regulation of the oocyte occurs from ovulation, when the oolemma detaches from the zona pellucida, and is known to be active only until compaction, which makes it a unique system of the early embryo [2]. Oocyte volume is determined by unique volume-specific regulatory mechanisms, which are involved in intracellular osmotic regulation, mostly based on efflux and influx of organic osmolytes such as glycine and betaine [2, 3]. Intracellular osmotic balance can be altered by the culture media composition and cryopreservation, which results in changes of oocyte volume and increases the risk of failed embryo development [1]. As a result, oocyte size markers, such as volume and area, are proposed as prognostic morphological markers for early embryo development and quality [4].

A decrease in oocyte volume, which can be caused by oocyte apoptosis or may result from dysregulation of volume-regulation mechanisms, increases the chances of embryonic developmental arrest [5]. Early animal studies confirm that the larger the oocyte size, the higher the developmental competence and blastulation rates [6, 7]. In humans, Weghofer et al. (2019) report that oocytes with larger oocyte area and oolemma diameter were more likely to fertilize and become cleavage-stage embryos of high score [8]. Additionally, embryos from oocytes with larger oolemma diameter had higher odds of being transferred or cryopreserved [8]. In the study by Bassil et al. (2021), embryos that developed from oocytes above or below the average size had lower good-quality blastulation rates [9]. Additionally, oocytes close to the mean oocyte diameter had higher odds of becoming a high quality day 5 blastocyst [9].

Based on current literature, there is not enough evidence to support that human oocyte size might be a non-invasive morphological marker and predictor of fertilization and early embryo development in both in vitro fertilization (IVF) and intracytoplasmic sperm injection (ICSI) treatment. Therefore, we aim to (i) describe human oocyte developmental dynamics from fertilization to the first cleavage division in used and discarded oocytes after IVF/ICSI treatment, and (ii) investigate the associations with embryo development, usage, and quality in order to establish a range of oocyte area as marker for early embryo development in clinical research.

## Methods

### Study design

This study is part of the Virtual EmbryoScope study, an ongoing subcohort study embedded in the Rotterdam Periconception Cohort (Predict study) [10]. Since 2009, the Predict study is an ongoing prospective tertiary hospital-based birth cohort study which takes place at the Department of Obstetrics and Gynecology of the Erasmus MC, University Medical Centre, the Netherlands. The Virtual EmbryoScope study was initiated in May 2017, with the aim to investigate the associations between the parental environment during periconception, fertility, and preimplantation embryonic growth in couples undergoing IVF treatment at the Erasmus MC. The study was approved by the Central Committee on Research in The Hague and the local Medical Ethics Committee of the Erasmus MC.

### Study population

Between May 2017 and July 2020, patients were eligible for enrollment in the study provided they had to undergo medically assisted reproduction due to male and/or female and/or unknown subfertility, were of ≥ 18 years of age, and could read and speak the Dutch language. Patients were included if they had autologous fresh oocytes that were fertilized by either IVF or ICSI with ejaculated or surgically retrieved testicular sperm, and their embryos were cultured in an EmbryoScope time-lapse incubator (EmbryoScope, Vitrolife, Sweden). Exclusion criteria were (i) embryo(s) not cultured in the time-lapse incubator, (ii) no fertilized oocytes available, and (iii) cases of oocyte donation or vitrification. All participants gave written informed consent before participation.

### Participant information

Information on the women and their male partners was obtained via self-administered questionnaires given at the time of enrollment (preconception), covering details such as age, education, lifestyle behavior and habits, and family and medical history. Additionally, at enrollment, anthropometric measurements including weight, height, and blood pressure were measured by qualified researchers. Information on subfertility diagnosis, assisted reproduction treatment, ovarian stimulation protocol, and clinical post-embryo transfer outcomes were retrieved from medical records. Fertilization rate for ICSI (per patient) was calculated according to the formula:

$$\mathrm{Fertilization}\;\mathrm{rate}=\frac{\mathrm{number}\;\mathrm{of}\;\mathrm{fertilized}\;\mathrm{oocytes}}{\mathrm{tota}\;\mathrm{lnumber}\;\mathrm{of}\;\mathrm{metaphase}\;\mathrm{II}\;\mathrm{oocytes}}$$

Embryo usage (per patient) was calculated according to the formula:

$$\mathrm{Embryo}\;\mathrm{usage}=\frac{\mathrm{number}\;\mathrm{of}\;\mathrm{usable}\;\mathrm{embryos}}{\mathrm{total}\;\mathrm{number}\;\mathrm{of}\;\mathrm{fertilized}\;\mathrm{oocytes}}$$

### Ovarian stimulation and oocyte retrieval

Women received routine ovarian stimulation protocols with either Menopur (hMG) or recombinant follicle-stimulating hormone (FSH; Bemfola, Gedeon Richter, Belgium, Menopur, Ferring, St. Prex, Switzerland, Gonal-F, Merck Serono, Switzerland or Rekovelle®, Ferring, St. Prex, Switzerland) with GnRH-agonist (Lucrin®, Abbott, Decapeptyl®, Ferring St. Prex, Switzerland) or GnRH-antagonist (Orgalutran®, MSD, Cetrotide®, Merck Serono, Fyremadel®, Ferring St. Prex, Switzerland) co-treatment protocol. A subcutaneous human recombinant chorionic gonadotropin (hCG) or GnRH-agonist (Ovitrelle®, Merck Serono, Switzerland, Pregnyl®, Organon, the Netherlands) injection was used for final follicular maturation. Oocytes were aspirated 35 h after ovulation induction and cultured in fertilization media (SAGE 1-step or G-TL Vitrolife).

### Fertilization methods

Ejaculated sperm was washed in commercially available discontinuous two-layer density gradient (45–90%, SpermGrad, Vitrolife), and testicular sperm was retrieved and frozen-thawed as previously described [11]. On the day of oocyte pick up, the total motile count was routinely determined after sperm processing by gradient density centrifugation. If after sperm processing a total motile sperm count of less than three million was recovered, fertilization was performed by ICSI, otherwise IVF was performed. Oocytes were fertilized according to routine IVF, ICSI with ejaculated sperm, or TESE-ICSI procedures as described previously [11]. Before ICSI, oocytes were denuded and only mature oocytes at the metaphase II stage were injected. Immature oocytes were discarded and excluded from all analyses and measurements. After sperm injection, oocytes were transferred to an EmbryoSlide (Vitrolife, Goteborg, Sweden) for culture in the time-lapse incubator. After insemination, IVF oocytes were cultured overnight and only fertilized dipronucleate oocytes were transferred to an EmbryoSlide and cultured in the EmbryoScope.

### Embryo culture, selection, and transfer

Oocytes were cultured in the EmbryoScope from t0 (time of fertilization) after ICSI and from tPNf (time of pronuclear fading) after IVF. Oocytes that were twice the normal volume (≥ 150 µM in diameter) are called “giant” oocytes, and these are routinely discarded before ICSI or after fertilization check in case of IVF due to their tetraploid origin [12]. These were not cultured in the EmbryoScope and therefore excluded from analyses and measurements. Culture media used were (i) SAGE 1-step (CooperSurgical) from June 2017 to December 2019 and (ii) G-TL (Vitrolife) from December 2019 to July 2020. Embryos were cultured at 36.8 °C, 7% O_2_, and 5% (SAGE 1-step) or 6% CO_2,_ (Vitrolife). Embryo selection for transfer was performed by morphological assessment at day 3 (July 2017–March 2019) or day 5 (April 2019–July 2020). Embryo selection for transfer was not aided by time-lapse information and was performed on a single image acquired by the EmbryoScope at 66–68 h (day 3) or 114–116 h (day 5) post-injection. Embryo morphology on day 3 was ranked according to the number of blastomeres, fragmentation, equality of blastomere size, and cell contact. Top ranking embryos contained eight blastomeres of equal size, with less than 10% fragmentation. Blastocysts were ranked by evaluating expansion, inner cell mass development, and trophectoderm appearance using the European Society of Human Reproduction and Embryology grading system [13]. All blastocysts were eligible for transfer. Embryo selection for cryopreservation was performed on day 4 (July 2017–March 2019) or day 5 (April 2019–July 2020), on a single image acquired by the EmbryoScope at 90–92 h or 114–116 h post-injection. Embryos with at least 13 blastomeres or showing at least 30% compaction were cryopreserved on day 4 [14]. On day 5, only blastocysts with at least a fair trophectoderm or inner cell mass were cryopreserved.

Single embryo transfer is part of standard protocols in our clinic; however, double embryo transfer was performed in cases of women ≥ 38 years of age or after two or more failed fresh IVF or ICSI cycles. Biochemical pregnancy was determined by an over the counter pregnancy test (urinary β-hCG test; Clearblue (SPD Swiss Precision Diagnostics, Geneva, Switzerland)) 10 days after transfer. Ongoing pregnancy was confirmed by the presence of a fetal heart beat during a transvaginal ultrasound examination at 12 weeks of gestation.

### Time-lapse imaging and oocyte measurements

**Fig. 1:**
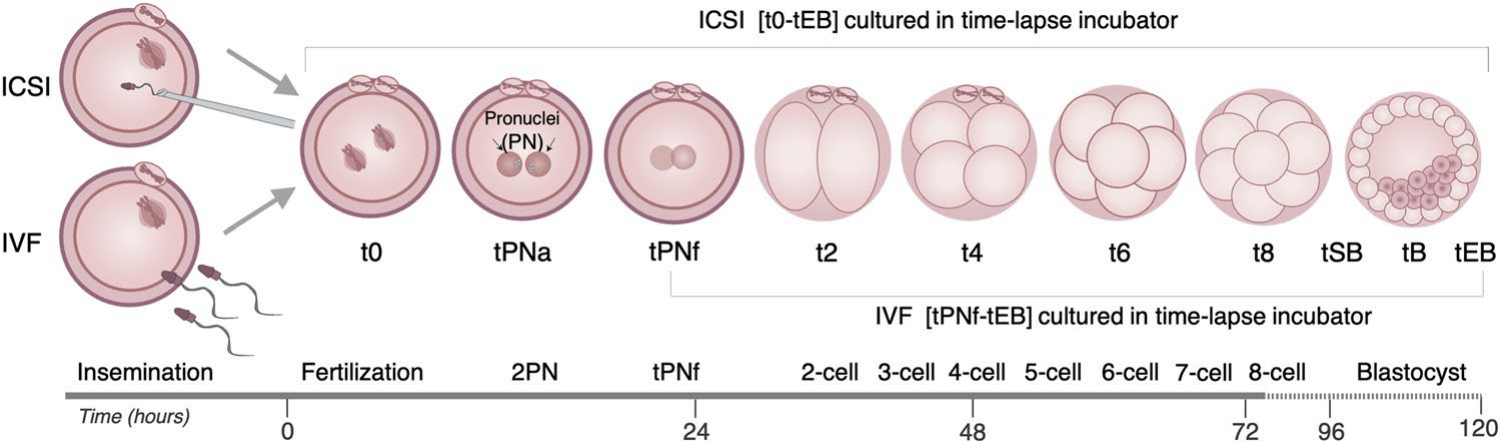
Schematic representation of embryo morphokinetics and annotations from fertilization to the blastocyst stage. In the order of time: t0, time of fertilization; tPNa, time of pronuclear appearance; tPNf, time of pronuclear fading; t2-–8-, time of cleavage to 2-, 3-, 4-, 5-, 6-, 7-, and 8-cells; tSB, start of blastulation; tB, full blastocyst stage and tEB, expanded blastocyst stage. ICSI, intracytoplasmic sperm injection; IVF, in vitro fertilization; PN, pronuclei

The EmbryoViewer software (Vitrolife) was used to manually perform oocyte measurements and annotate embryo morphokinetics according to the ESHRE guidelines for dynamic monitoring of human preimplantation development [15]. For embryos that were selected for transfer or cryopreservation, we recorded the timing of fertilization (t0), pronuclear appearance (tPNa), and fading (tPNf), as well as the timing of reaching the 2-, 3-, 4-, 5-, 6-, 7-, 8-, 9-cell stage (t2, t3, t4, t5, t6, t7, t8, t9), the start of blastulation (tSB), and reaching the full (tB) and expanded blastocyst stage (tEB) (Fig. 1) [16]. At tEB, the inner cell mass (ICM) and the trophectoderm (TE) were graded A, B, or C according to the blastocyst grading system developed by Gardner and Schoolcraft [17]. t0, tPNa, and tPNf were additionally annotated for discarded embryos, whereas tPNa and tPNF were only available for the fertilized discarded embryos.

**Fig. 2:**
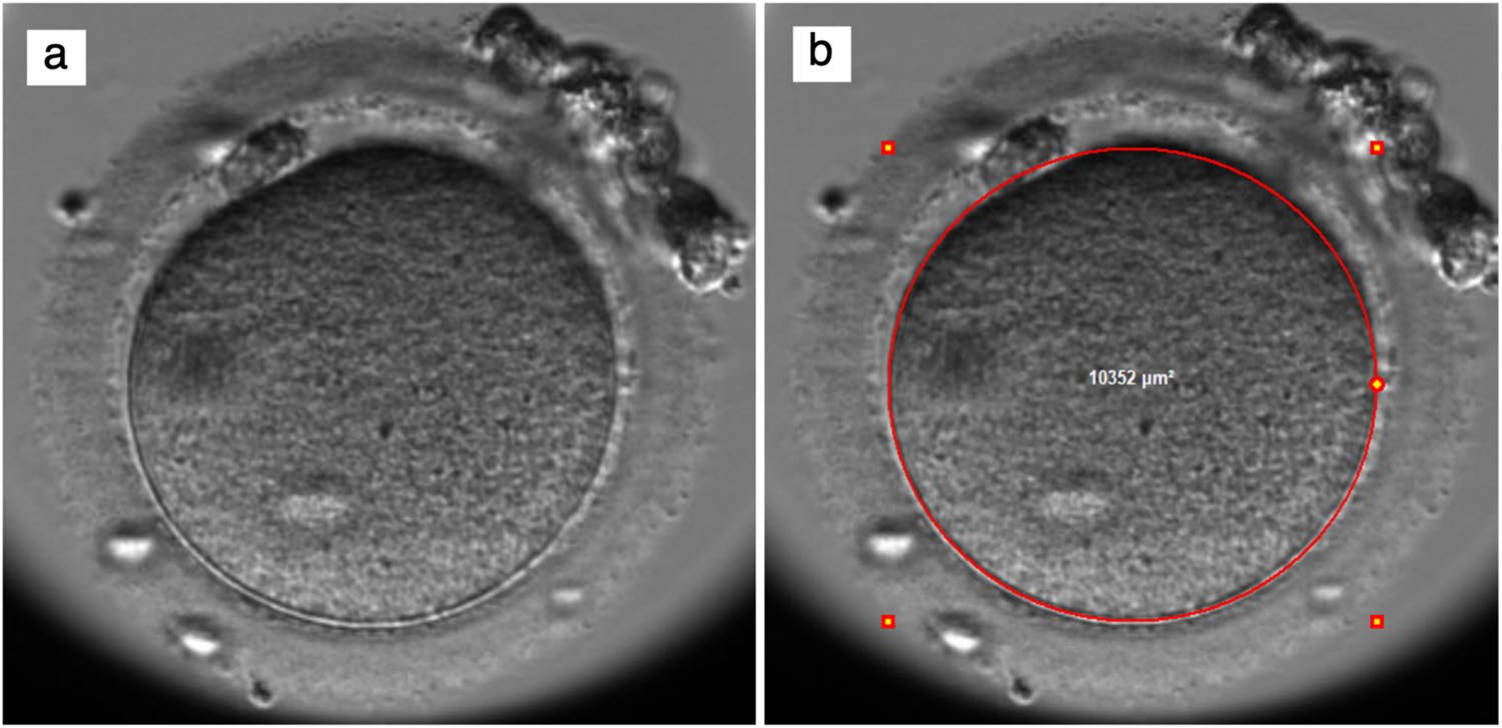
Oocyte area performed with the ellipse tool of the Embryoviewer software (Vitrolife) at t0. The red circle delineates the outermost perimeter of the oocyte, excluding the zona pellucida and the perivitelline space. (**a**) Before and (**b**) after oocyte area measurement

Oocytes were termed “used” if they were fertilized, and embryo development resulted in an embryo that was cryopreserved or transferred, and otherwise termed “discarded.” Discarded oocytes were classified as either fertilized (2PN or abnormal PN), unfertilized or unmeasurable. The term “oocyte area” refers to the area of the oocyte excluding the zona pellucida. Oocyte area was measured with the ellipse tool of the Embryoviewer software (Vitrolife) at t0, tPNa, and tPNf in ICSI oocytes and only at tPNf for IVF oocytes (Fig. 2), where t0 is the time of fertilization specifically referring to the time of injection of the last oocyte during ICSI. Out of the standard seven focal plane pictures, the focal plane with the clearest ooplasm perimeter was selected for measuring.

### S*tatistical analysis*

Baseline characteristics are depicted as mean and standard deviation (SD), median and interquartile range (IQR), or number and percentage. Comparison of baseline characteristics between groups was performed with a Student’s *t* test in case of normally distributed data or Mann–Whitney *U* test for non-normally divided continuous data and Chi-square or Fisher’s exact test for categorical variables. All analyses were performed with oocyte area as continuous variable. Oocyte area at t0, tPNa, and tPNf are studied by using a linear mixed effects model to account for the clustering of multiple oocytes of one couple and using the difference in oocyte area between the timepoints (t0-tPNa and tPNa-tPNf) as response variables to account for paired data.

Each oocyte follows its own developmental pattern resulting in a different timing of pronuclei appearance and fading. Therefore, we calculated the shrinking rate of oocyte area by using a linear regression model to summarize the individual trajectory of oocyte area (at tPNa and tPNf) per oocyte, with timing of t0, tPNa, and tPNf as independent variable. Subsequently, to study if oocyte area at fertilization (t0) is associated with oocyte shrinking a linear mixed is used.

Differences in oocyte area and shrinking rate between fertilization method (IVF or ICSI), fertilization, PN occurrence, and embryo usage (used or discarded) were additionally studied with a mixed effects model.

A mixed effects logistic regression model was used to study the association between oocyte area, fertilization, and the embryo usage, using the GLMM adaptive package in R. This model is used for dichotomous data, such as fertilization (yes or no) and embryo usage (used or discarded), and takes into account patient-specific intercepts regarding the correlation between sibling oocytes. Random subject effects are used in this model to account for the clustering of multiple oocytes of one couple. Subsequently, the oocyte area at t0 and tPNf was divided into deciles to compare, respectively, the significance between the frequency of fertilized oocytes and used embryos between deciles using the Chi-square test. Deciles were: D1 (7180–9630 µm^2^), D2 (9630 µm^2^–9940 µm^2^), D3 (9940–10,100 µm^2^), D4 (10,100–10,300 µm^2^), D5 (10,300–10,500 µm^2^), D6 (10,500–10,700 µm^2^), D7 (10,700–10,800 µm^2^), D8 (10,800–11,100 µm^2^), D9 (11,100–11,500 µm^2^), and D10 (11,500–14 000 µm^2^).

Analyses on the developmental time points of reaching the different cell stages were performed solely on the transferred and cryopreserved embryos. Spearman rho rank correlation coefficient (*R*) was used to evaluate correlations between oocyte area at tPNf and embryo morphokinetics in hours. Linear mixed models were applied to study the association between oocyte area at tPNf or shrinking rate and embryo morphokinetics in minutes (tPNf, t2, t3, t4, t5, t6, t7, t8, t9, tSB, tB, and tEB). A mixed effects model was used to study differences in oocyte area at tPNf among the ICM and TE groups.

All models were reported as crude in the absence of confounder adjustment, and adjusted for maternal age, ovarian stimulation protocol, sperm retrieval method, and culture medium [18].

The time-points t0 and tPNa were recoded solely for embryos fertilized with ICSI, but not IVF, as fertilization does not occur under the microscope, and its timing is therefore estimated by the embryologist. Consequently, embryos fertilized with IVF were excluded from the analyses regarding fertilization and embryo usage, pre-implantation embryo morphokinetics, and post-implantation treatment outcomes. To study and describe human oocyte developmental dynamics from fertilization to the first cleavage divisions in used and discarded oocytes as a biological phenomenon and not solely due to the ICSI procedure, we included IVF oocytes to compare oocyte area between IVF and ICSI oocytes at time-point tPNf.

Since from the used embryos, the largest number of oocyte measurements are available at tPNf, the analyses involving only used embryos (embryo morphokinetics and post-implantation treatment outcomes) were performed with oocyte area at tPNf.

Two-sided *p* values < 0.05 were considered statistically significant. All analyses were performed in R (R for Windows, version 3.6.2 R Core Team).

## Results

### Baseline characteristics

**Fig. 3:**
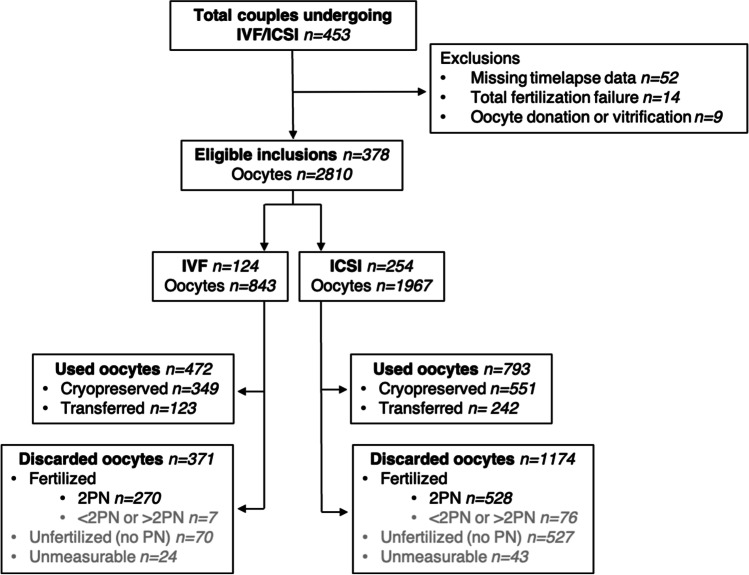
Flowchart of participant inclusion and exclusion criteria (May 2017–July 2020). Eligible inclusions were stratified by fertilization method. Oocytes were termed “used” if they were fertilized, and embryo development resulted in an embryo that was cryopreserved or transferred, or otherwise “discarded.” Discarded oocytes were classified as either fertilized, unfertilized, or unmeasurable. Grey text indicates the discarded oocytes excluded from the main analyses. ICSI, intracytoplasmic sperm injection; IVF, in vitro fertilization; PN, pronuclei

A total of 453 women were enrolled in the Virtual EmbryoScope study from May 2017 to July 2020. Women who had missing time-lapse data (*n* = 52), total fertilization failure (*n* = 14), or oocyte donation or vitrification (*n* = 9) were excluded (Fig. 3). This resulted in 378 IVF/ICSI cycles finally analyzed in the study, with in total 2810 retrieved oocytes. Baseline characteristics of the included and excluded population are compared in Table S1. Stratification for fertilization method resulted in 33% (*n* = 124) IVF and 67% (*n* = 254) ICSI treatments. In case of IVF, 843 oocytes were retrieved, of which 56% (*n* = 472) were used, and 44% (*n* = 371) were discarded. For ICSI, 1967 oocytes were retrieved, of which 40% (*n* = 793) resulted in an embryo that was used for transfer of cryopreservation, and 60% (*n* = 1174) were discarded. Fertilization method was significantly associated with the number of used and discarded oocytes, in favor for IVF (*p* < 0.001). Baseline characteristics of the included population stratified by fertilization method are represented in Table 1. Women undergoing IVF were older than those undergoing ICSI. Fertilization method was associated with subfertility diagnosis, the number of embryos transferred, and the days of embryo culture. Women undergoing IVF had a higher fertilization rate and a higher number of total usable embryos compared to those undergoing ICSI.

**Table 1:** Baseline characteristics of the included population stratified by fertilization method

	Total study participants *n* = 378^1^	ICSI *n* = 254^1^	IVF *n* = 124^1^	*p* value
Maternal characteristics				
Maternal age, years	33.7 ± 4.9	33.0 ± 4.7	35.2 ± 4.8	< 0.001*
Nulliparous	282 (74.6)	197 (77.6)	85 (68.5)	0.08
Geographic origin				0.18
Dutch	290 (79.0)	202 (81.8)	88 (73.3)	
Western	17 (4.6)	10 (4.0)	7 (5.8)	
Non-Western	60 (16.3)	35 (14.2)	25 (20.8)	
Education				0.55
Low	21 (5.7)	16 (6.5)	5 (4.1)	
Middle	146 (39.6)	100 (40.3)	46 (38.0)	
High	202 (54.7)	132 (53.2)	70 (57.9)	
Maternal BMI (kg/m^2^)	24.7 [21.9, 27.8]	24.9 [22.4, 27.6]	23.9 [21.3, 28.1]	0.15
BMI > 25	175 (46.4)	122 (48.0)	53 (43.1)	
BMI > 30	63 (16.7)	43 (16.9)	20 (16.3)	
Mean arterial pressure, *at study entry*	86.0 [79.3, 93.3]	87.3 [80.0, 94.0]	84.3 [78.6, 91.4]	0.03*
Folic acid supplement use	345 (93.5)	230 (92.7)	115 (95.0)	0.61
Multivitamin use	186 (54.5)	121 (52.6)	65 (58.6)	0.36
Alcohol use	157 (42.9)	106 (43.1)	51 (42.5)	0.78
Smoking	50 (13.7)	33 (13.4)	17 (14.2)	0.77
Treatment factors				
Subfertility diagnosis				< 0.001*
Only male factor	169 (44.7)	164 (64.6)	5 (4.0)	
Only female factor	86 (22.8)	4 (1.6)	82 (66.1)	
Combined	80 (21.2)	77 (30.3)	3 (2.4)	
Unexplained	42 (11.1)	8 (3.1)	34 (27.4)	
PCOS	76 (20.1)	46 (18.1)	30 (24.2)	0.21
Fertilization method, ICSI	254 (67.2)			
ICSI with ejaculated sperm	145 (38.4)			
ICSI with surgically retrieved sperm	109 (28.8)			
Ovarian stimulation, GnRH-agonist	102 (27.2)	63 (24.9)	39 (32.0)	0.19
No. of embryos transferred				0.002*
No ET	18 (4.8)	12 (4.7)	6 (14.8)	
Single ET	312 (82.5)	222 (87.4)	90 (72.6)	
Double ET	48 (12.7)	20 (7.9)	28 (22.6)	
Days of culture				0.008*
3	255 (67.5)	160 (63.2)	95 (79.2)	
5	117 (30.9)	92 (36.4)	25 (20.8)	
Culture media				0.14
SAGE 1-Step	329 (87.0)	216 (85.0)	113 (91.1)	
GTL Vitrolife	49 (13.0)	38 (15.0)	11 (8.9)	
Treatment outcomes				
Oocytes aspirated	8 [5, 13]	8 [6, 13]	8 [5, 12]	0.64
Total fertilized oocytes	5 [3, 7]	4 [3, 7]	5 [3, 8]	0.05
Fertilization rate	na	0.85 [0.43, 0.71]	na	na
Total usable embryos	3 [2, 5]	2 [2, 4]	3 [2, 5]	0.001*
Embryo usage	0.67 [0.50, 0.86]	0.67 [0.50, 0.88]	0.67 [0.50, 0.85]	0.17
OHSS	26 (6.9)	16 (6.3)	10 (8.1)	0.15
hCG +	147 (43.2)	105 (41.3)	42 (33.9)	0.49
GS +	133 (60.9)	93 (36.6)	37 (29.8)	0.39
Fetal heart beat +	120 (64.7)	86 (33.9)	34 (27.4)	0.53
Live birth	111 (34.9)	81 (31.9)	30 (24.2)	0.33

Data is presented as mean ± standard deviation, median [interquartile range], or number of individuals (percentage). *Significant differences between IVF and ICSI are reported with a *p* < 0.05. ^1^Missing data rates by variable were reported and excluded from the total sum of participants or the denominator during analyses. *BMI*, body mass index; *ET*, embryo transfer; *GnRH*, gonadotropin releasing hormone; *GS*, gestational sac; *hCG*, human chorionic gonadotropin; *ICSI*, intracytoplasmic sperm injection; *IVF*, in vitro fertilization; *na*, not applicable; *OHSS*, ovarian hyperstimulation syndrome; *PCOS*, polycystic ovary syndrome

### Oocyte dynamics from fertilization to pronuclear fading

**Fig. 4:**
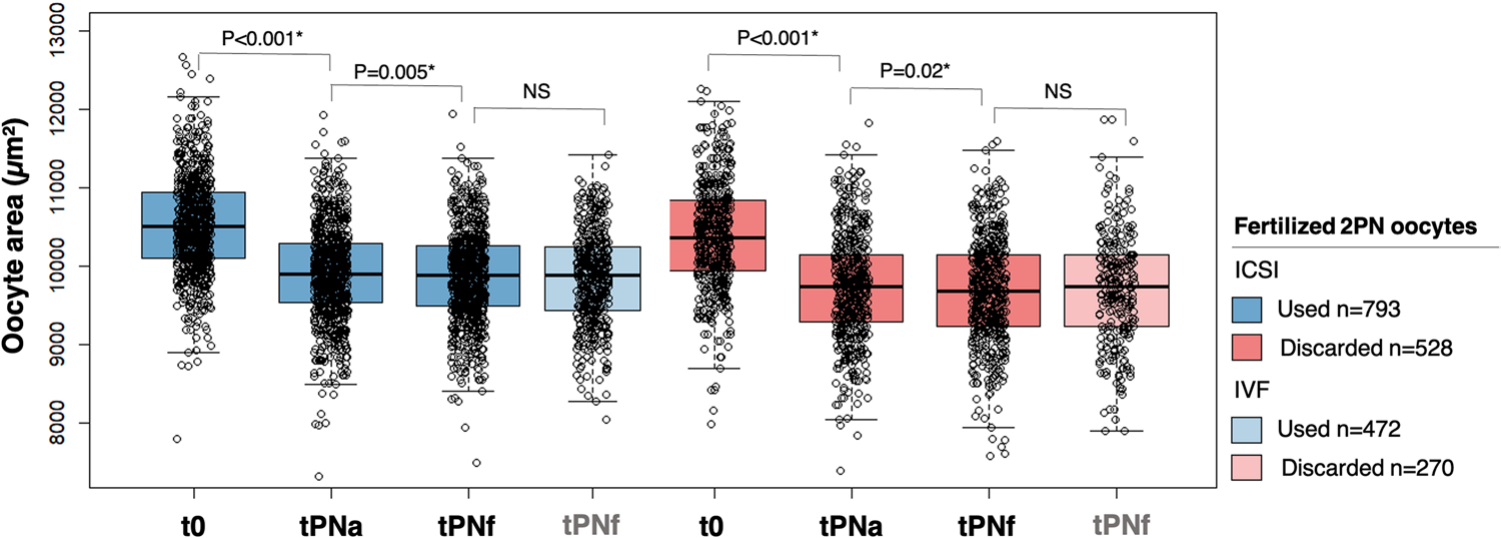
Box and whisker plots of longitudinally measured oocyte area at t0, tPNa, and tPNf in used ICSI oocytes (*n* = 793) [blue] and in fertilized (2PN) discarded ICSI oocytes (*n* = 528) [pink]. Oocyte area is measured at tPNf in used IVF oocytes (*n* = 472) [light blue] and in fertilized (2PN) discarded IVF oocytes (*n* = 270) [light pink]. Boxplots present median, 10th, 25th, 75th, and 90th percentiles. Oocyte area is compared among the time-points and compared among ICSI and IVF at tPNf by a mixed effects model to account for the clustering of multiple embryos of one couple in the model. *Significant differences with a *p* < 0.05. ICSI, intracytoplasmic sperm injection; IVF, in vitro fertilization; NS, non-significant; t0, time of fertilization; tPNa, time of pronuclear appearance; tPNf, time of pronuclear fading

To study changes in oocyte area over time, oocyte area of used and discarded oocytes was measured and compared at t0, tPNa, and tPNf after ICSI (Fig. 4). Oocyte area significantly decreased from t0 to tPNa in used (oocyte area at t0 10,519 µm^2^ ± 624 versus tPNa 9898 µm^2^ ± 614, *p* < 0.001) and discarded oocytes (oocyte area at t0 10,379 µm^2^ ± 677 versus tPNa 9730 µm^2^ ± 681, *p* < 0.001), and from tPNa to tPNf in used (oocyte area at tPNa 9898 µm^2^ ± 614 versus tPNf 9864 µm^2^ ± 595, *p* = 0.005) and discarded oocytes (oocyte area at tPNa 9730 µm^2^ ± 681 versus tPNf 9679 µm^2^ ± 673, *p* = 0.02). The mean oocyte area shrinking rate from t0 to tPNf was 25.9 µm^2^/h ± 23.8 for used oocytes and 26.1 µm^2^/h ± 24.1 for discarded oocytes. Oocyte area at fertilization (t0) was positively associated with oocyte area shrinking rate both for used and discarded oocytes (t0-tPNf), as the larger the oocyte area at t0, the faster the shrinking rate (Table 2). Oocyte area at tPNf did not differ between ICSI and IVF oocytes both in used and discarded oocytes (*P* = NS) (Fig. 4).

**Table 2:** Association between oocyte area after ICSI at t0 and oocyte area shrinking rate

		Oocyte area (10^–3^ µm^2^)			
		Crude model		Adjusted model	
		Beta (95%CI) µm^2^/h	*p* value	Beta (95%CI) µm^2^/h	*p* value
Used	*n* = 793	− 12.422 (− 14.418; − 10.425)	< 0.001*	− 12.551 (− 14.550; − 10.553)	< 0.001*
Discarded	*n* = 528	− 12.084 (− 14.733; − 9.435)	< 0.001*	− 12.041 (− 14.730; − 9.352)	< 0.001*

Discarded oocytes include those fertilized with 2PN. Crude model is adjusted for time of oocyte area measurement, the adjusted model additionally for maternal age, ovarian stimulation method, sperm retrieval method, and culture medium. *Significant differences with a *p* < 0.05. *CI*, confidence interval; *ICSI*, intracytoplasmic sperm injection; *PN*, pronuclei; *t0*, time of fertilization

### Oocyte dynamics of used and discarded oocytes

In this study, discarded oocytes included oocytes with no PN, < 2PN, 2PN, > 2PN, and only the fertilized 2PN were chosen to be compared with the used oocytes in the main analyses (Fig. 3). Oocyte area from fertilization to pronuclear fading differed between used and discarded oocytes, as shown in Table 3. Oocyte area of oocytes that developed into used embryos was significantly larger at t0, tPNa, and tPNf, and the oocytes reached tPNa and tPNf faster than discarded oocytes (Table 3). Oocyte area shrinking rate did not differ between used and discarded oocytes (Table 3). Since maternal age may affect oocyte developmental competence, we performed an additional analyses with adjustments for maternal age at study intake. However, when adjusting the models for maternal age, all differences between used and discarded oocytes remained unaltered (data not show).

**Table 3:** Longitudinal measurements of oocyte area after ICSI stratified for used and discarded (fertilized 2PN) embryos

		Used oocytes Mean ± SD *n* = 793	Discarded oocytes Mean ± SD *n* = 528	*p* value
Oocyte area (µm^2^)	t0	10,519 ± 624.5	10,379 ± 677	< 0.001*
	tPNa	9898 ± 614	9730 ± 681	< 0.001*
	tPNf	9864 ± 595	9679 ± 673	< 0.001*
Time (h)	tPNa	7.7 ± 2.3	9.3 ± 5.0	< 0.001*
	tPNf	23.6 ± 3.2	25.6 ± 5.9	< 0.001*
Shrinking rate (µm^2^/h)		− 25.9 ± 23.8	− 26.1 ± 24.1	0.517

Measurements are performed at t0, tPNa and tPNf in ICSI oocytes only (*n* = 1321). Data is presented as mean ± standard deviation, and groups are compared by using a linear mixed effects model. *Significant differences with a *p* < 0.05. *ICSI*, intracytoplasmic sperm injection; *PN*, pronuclei; *SD*, standard deviation; *t0*, time of fertilization; tPNa, time of pronuclear appearance; *tPNf*, time of pronuclear fading

Differences between discarded oocytes that were fertilized and unfertilized and oocytes with 2PN and < 2PN or > 2PN are described in Tables S2 and S3. Here, we found that unfertilized oocytes were significantly larger compared to fertilized oocytes at t0. No differences were found between 2PN oocytes and oocytes with an abnormal number PN.

### Oocyte area and preimplantation embryo development

Oocyte area at t0 was negatively associated with fertilization, as a larger oocyte area had a lower odds of being fertilized (2PN) (range 7183 to 14,000 µm^2^) (Table 4). Subsequently, we found that in the fertilized oocytes (range 7120 to 11,930 µm^2^), oocyte area was positively associated with embryo usage both at t0, tPNa, and tPNf, as a larger oocyte area had a higher odds of being transferred or cryopreserved than to be discarded (e.g., oocyte area at t0: adjusted OR_embryo usage_ 1.58, 95%CI 1.27, 1.95, *p* < 0.001). (Table 4). Removing day 5 embryos from the analysis did not change the results on embryo usage.

**Table 4:** Mixed effects logistic regression model of oocyte area after ICSI, and fertilization and embryo usage

		Fertilization yes/no Crude model			Fertilization yes/no Adjusted model		
		Estimate	OR (95%CI)	*p* value	Estimate	OR (95%CI)	*p* value
Oocyte area (10^−3^ µm^2^)	t0	− 0.573	0.564 (0.479; 0.665)	< 0.001*	− 0.564	0.570 (0.484; 0.670)	< 0.001*
		Used/discarded Crude model			Used/discarded Adjusted model		
		Estimate	OR (95%CI)	*p* value	Estimate	OR (95%CI)	*p* value
Oocyte area (10^−3^ µm^2^)	t0	0.461	1.586 (1.284; 1.959)	< 0.001*	0.455	1.576 (1.274; 1.951)	< 0.001*
	tPNa	0.581	1.788 (1.430; 2.235)	< 0.001*	0.582	1.789 (1.429; 2.240)	< 0.001*
	tPNf	0.513	1.670 (1.338; 2.084)	< 0.001*	0.512	1.669 (1.336; 2.085)	< 0.001*

A mixed effects logistic regression model is used to analyze the association between oocyte area and fertilization (2PN) and embryo usage (odds to develop into a used oocyte). The model of fertilization (2PN) is performed in the ICSI group with t0 measurements of 1321 fertilized (2PN) and 527 unfertilized oocytes (range 7183 to 14,000 µm^2^). Prediction of embryo usage is performed in the ICSI group with 793 used oocytes and 528 discarded oocytes (range 7120 to 11,930 µm^2^). Crude model is adjusted for time of oocyte area measurement, the adjusted model additionally for maternal age, ovarian stimulation method, sperm retrieval method, and culture medium. *Significant differences with a *p* < 0.05. *ICSI*, intracytoplasmic sperm injection; *OR*, odds ratio; *PN*, pronuclei; *t0*, time of fertilization; *tPNa*, time of pronuclear appearance; *tPNf*, time of pronuclear fading

After dividing oocyte area at t0 into deciles, we found that the percentage of oocytes that fertilized (2PN) was between 67 and 82% in all deciles except the uppermost decile, that comprised 45% of the oocytes that fertilized, *p* < 0.001. Therefore, we repeated the association analyses between oocyte area at t0 and fertilization success removing those oocytes from the uppermost decile and found a positive but non-significant association (oocyte area at t0: adjusted OR_fertilization_ 1.13, 95%CI 0.89; 1.43, *p* = 0.31). We also found that the percentage of oocytes that developed into used embryos ranged from 67 to 87% in all deciles except the lowest decile (D1), that comprised 45% of oocytes that were used, *p* = 0.007. The same was found at tPNf, *p* < 0.001.

Linear mixed modelling after adjusting for confounders (maternal age, ovarian stimulation method, sperm retrieval method, and culture medium) reported that, in used oocytes (range 7120 to 11,930 µm^2^), larger oocyte area at tPNf associated with faster embryo development (Table 5). Specifically, embryos with a larger oocyte area at tPNf were significantly faster at developing to the t2-, t3-, t4-, t5-, t6-, t7-, and t9-cell stage (e.g., adjusted β_t9_ − 0.131 min, 95%CI − 0.237, − 0.025, *p* = 0.016). As example, retransformation of the betas to the original values showed that for every increase in oocyte area of 200 µm^2^, the embryo reached t9 0.44 h earlier.

**Table 5:** Linear mixed effect model between oocyte area at tPNf in ICSI used oocytes (*n* = 793, range 7120 to 11,930 µm^2^) and embryo morphokinetics

			Oocyte area at tPNf (µm^2^)			
Embryo developmental time-points (minutes)	Patient number	Embryo number	Crude		Adjusted	
			Beta (95%CI)	*p* value	Beta (95%CI)	*p* value
tPNf	251	780	− 0.050 (− 0.072, − 0.028)	< 0.001*	− 0.048 (− 0.070, − 0.026)	< 0.001*
t2	251	780	− 0.049 (− 0.074, − 0.026)	< 0.001*	− 0.049 (− 0.073, − 0.025)	< 0.001*
t3	251	778	− 0.069 (− 0.105, − 0.033)	< 0.001*	− 0.064 (− 0.099, − 0.028)	< 0.001*
t4	251	776	− 0.063 (− 0.101, − 0.026)	< 0.001*	− 0.061 (− 1.00, − 0.023)	< 0.001*
t5	247	769	− 0.145 (− 0.199 − 0.091)	< 0.001*	− 0.142 (− 0.196 -0.087)	< 0.001*
t6	245	758	− 0.105 (− 0.156, − 0.054)	< 0.001*	− 0.106 (− 0.157, − 0.054)	< 0.001*
t7	239	734	− 0.076 (− 0.132, − 0.019)	< 0.001*	− 0.082 (− 0.140, − 0.025)	< 0.001*
t8	225	673	− 0.017 (− 0.083, 0.049)	0.146	− 0.030 (− 0.096, 0.037)	0.381
t9	106	202	− 0.139 (− 0.243, − 0.034)	0.010*	− 0.131 (− 0.237, − 0.025)	0.016*
tSB	92	264^a^	0.043 (− 0.049, 0.135)	0.355	0.044 (− 0.051, 0.138)	0.365
tB	86	238^a^	0.040 (− 0.052, 0.132)	0.388	0.046 (− 0.049, 0.141)	0.341
tEB	78	225^a^	0.045 (− 0.051, 0.142)	0.353	0.043 (− 0.056, 0.142)	0.397

Linear mixed effect models were applied to study the association between oocyte area in µm^2^ and preimplantation developmental timing measures in minutes. Model is adjusted for maternal age, ovarian stimulation method, sperm retrieval method, and culture medium. ^a^Until April 1, 2019, embryo transfer was performed on day 3 after fertilization; thereafter, embryos are incubated till day 5 after oocyte retrieval and tSB, tB, and tEB parameters could be assessed. *Significant differences with a *p* < 0.05. *CI*, confidence interval; *ICSI*, intracytoplasmic sperm injection; *tPNf*, time of pronuclear fading; *t2–t9*, time of cleavage to 2- till 9-cells; *tSB*, start of blastulation; *tB*, full blastocyst stage; *tEB*, expanded blastocyst stage

**Fig. 5:**
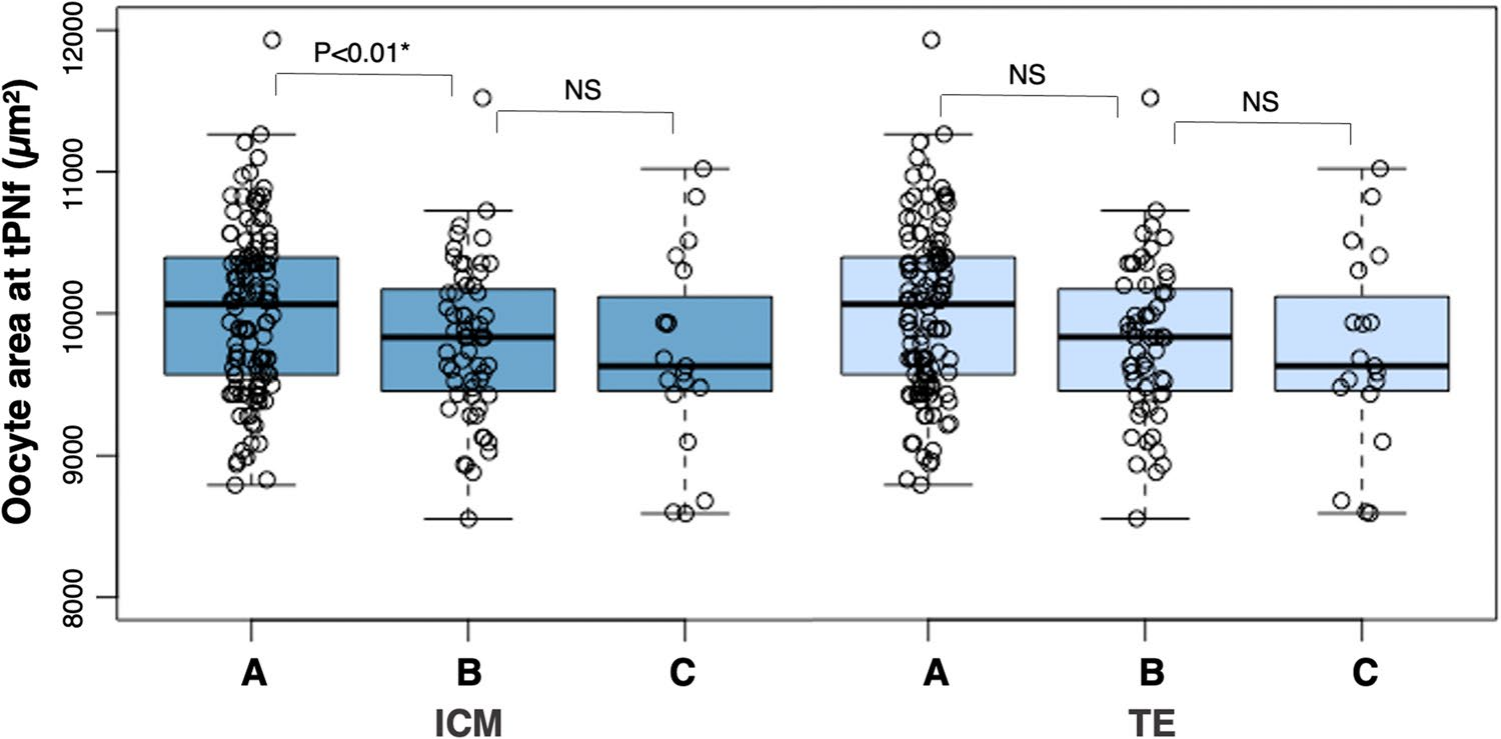
Box and whisker plots of oocyte area measured at tPNf in used ICSI embryos incubated until day 5 after fertilization grouped by ICM (*n* = 201) and TE (*n* = 199). ICM morphology is graded as A (*n* = 124), B (*n* = 61), and C (*n* = 19) [dark blue] and the TE as A (*n* = 88), B (*n* = 81), and C (*n* = 33) [light blue]. Boxplots present median, 10th, 25th, 75th, and 90th percentiles. Oocyte area measured at tPNf is compared among the ICM and TE groups by a mixed effects model to account for the clustering of multiple embryos of one couple in the model. *Significant differences with a *p* < 0.05. ICM, inner cell mass; ICSI, intracytoplasmic sperm injection; NS, non-significant; TE, trophectoderm; tPNf, time of pronuclear fading

Oocytes that developed into a blastocyst with grade A ICM were significantly larger at tPNf (10,008 µm^2^ ± 582) compared to those that developed a grade B (9811 µm^2^ ± 538) or C (9721 µm^2^ ± 696) ICM (*p* < 0.01) (Fig. 5). For TE, oocyte area did not differ significantly among the grades A, B, or C.

## Discussion

In this study, we show that oocyte area is associated with preimplantation embryo usage, quality, and development. In summary, we found that oocyte area shrinks from fertilization to pronuclear fading, and the larger the oocyte area at t0, the faster the shrinking rate. Oocyte area was larger in used compared to discarded oocytes. A larger oocyte at t0 had lower odds of fertilizing, specifically if the oocyte area was in the uppermost decile (11,500–14,000 µm^2^). Among the fertilized oocytes (range 7120 to 11,930 µm^2^), the odds of an embryo being transferred or cryopreserved increased with increasing oocyte area. Larger oocyte area at tPNf was associated with faster preimplantation embryo development and higher quality ICM.

This is the first study to describe oocyte area dynamics prior to pronuclear fading. We report that oocyte area decreases significantly before the first cleavage division and hypothesize; it occurs due to the cytoskeletal reorganization that takes place during pronuclear appearance and fading, and in preparation for cytokinesis [19]. Oocyte area shrinking occurred most likely for both IVF and ICSI oocytes, as no differences in oocyte area at tPNf were found. Which explains that oocyte shrinking after fertilization is most likely a biological phenomenon and not due to the ICSI procedure [20]. We did not find any associations with embryo usage or embryo morphokinetics, so oocyte area shrinking may be a biological mechanism unrelated to embryonic competence.

Our results are in agreement with and expand the current knowledge on the role of oocyte size and embryo development [8, 9]. We describe that larger oocytes at t0, tPNa, and tPNf with a range of 7120 to 11,930 µm^2^ are more likely to develop into cryopreserved or transferred embryos, and that larger oocytes at tPNf result in embryos with higher quality ICM, and develop faster for the stages of symmetric cell divisions. During the period of oogenesis and folliculogenesis, the oocyte is exposed to environmental cues that support its metabolic maturation, gene activation, protein synthesis, and cytoskeletal reorganization, which are manifested by cellular hypertrophy. This has been demonstrated by animal studies, where oocyte size was correlated to the accumulation of maternal transcripts and metabolic activity [21]. For example, transcripts of DNA methyltransferases, involved in the maternal epigenetic regulation of the embryonic genome [21], and amino acid-based transporters [3]. In the study by Hiura et al. (2006), mice oocytes showed size-dependent-methylation activity, where DNA methylation progressed according to oocyte growth [22]. This was consistent with human studies, as Leary et al. (2015) report that oocytes of overweight and obese women compared to normal-weight controls were small, manifested altered glucose consumption, amino acid profiles, and triglyceride levels, and were less likely to reach the blastocyst stage [23]. Therefore, we hypothesize that a larger oocyte area more likely reflects a matured oocyte which can metabolically and epigenetically support the first stages of embryo development. Indeed, we find that a larger oocyte area is associated with faster embryo development, until maternal transcripts are gradually replaced by the embryonic genome, and the embryo changes its main source of energy from pyruvate to glucose.

It was unexpected to find that oocyte area had an inverse association with fertilization success, given the opposite trend in the following analyses. We hypothesized that the negative association was driven by the oocytes present in the uppermost decile of oocyte area, since those oocytes were excluded from the following analyses as they did not fertilize. Oocytes damaged by ICSI commonly swell before it becomes apparent that they degenerate. Indeed, after removing oocytes in the uppermost decile, the association with fertilization success became positive although not significant.

According to recent literature, faster embryo morphokinetics favor successful pregnancy outcomes. Fast blastulation is predictive of achieving a positive clinical pregnancy, ongoing pregnancy, and live birth compared to slower-developing embryos [24–26]. However, we did not see such an association (Table S4) as we hypothesize that later stages of implantation are more likely influenced by maternal health factors, hormonal stimulation, and the intrauterine environment.

Oocyte area hold potential as non-invasive marker for embryo usage at a very early stage in development. Oocyte area therefore could be integrated within the current morphological scoring systems for early embryos and used in circumstances where embryo selection cannot occur at the blastocyst stage.

To our knowledge, this is the first study with a large sample size analyzing longitudinal measurements of oocyte area and its association with both pre- and post-transfer clinical treatment outcomes. The use of extensive questionnaires and standardized measurements reinforced the reliability of the results. Potential residual confounding may account for a study limitation. Additionally, we could not compare oocyte area and embryo morphokinetics between IVF and ICSI, as IVF oocytes were not cultured in the EmbryoScope incubator prior to tPNf, and embryos could not be annotated from the time of fertilization as for ICSI. A larger sample size beyond the morula stage would have been advantageous to increase reliability. Lastly, we could only hypothesize causal explanations as no molecular data could be obtained to sustain evidence found using morphological data. Therefore, future studies should incorporate analysis of metabolites secreted from the oocyte in culture media.

We suggest further research on maternal factors that may affect oocyte area, such as maternal health and infertility, along with the question whether oocyte size can be a non-invasive marker part of current selection criteria in assisted reproduction. We propose elaborate research on polycystic ovary syndrome, ovarian stimulation protocols, and ovarian hyperstimulation syndrome, or (non)fertility-related conditions, for example, advanced biological age or auto-immune diseases, that affect the ovarian microenvironment, as the role of oocyte area and embryo development may differ for such patients. This will bring us to investigate which determinants of oocyte quality and embryo development should take center stage in developing preconception care strategies. With regard to morphological data, we suggest research on the role of the follicle (size, cells, and follicular-oocyte maturation signals) on oocyte maturation and size. Subsequently, in vitro maturation (IVM) of oocytes also provides the opportunity to study nuclear and cytoplasmic maturation during in vitro conditions. In addition, future investigation should also address paternal contributions to preimplantation embryo development. Male gamete contributions to early embryo development are a complex of genetic and non-genetic components that determine embryogenesis after fertilization.

## Conclusion

This study highlights the relevance of oocyte morphology in assisted reproduction and how embryo usage is associated to oocyte area. We found that a larger oocyte area is associated with the odds of having a cryopreserved or transferred embryo and is associated with embryo morphokinetics and quality, but not with clinical treatment outcomes. Oocyte area may be an early marker in clinical research to predict the success of embryo development.

## Supplementary Information

Below is the link to the electronic supplementary material.

Supplementary file1 (DOCX 32 KB)

## Data Availability

The data underlying this article will be shared upon reasonable request to the corresponding author.

## Abbreviations

ART: Assisted reproductive treatment
BMI: Body mass index
ET: Embryo transfer
FHB: Fetal heart beat
FSH: Follicle-stimulating hormone
GnRH: Gonadotropin-releasing hormone
GS: Gestational sac(s)
hCG: Human chorionic gonadotropin
hMG: Human menopausal gonadotropin
ICM: Inner cell mass
ICSI: Intracytoplasmic sperm injection
IQR: Interquartile range
IVF: In vitro fertilization
LB: Live birth
Na: Not applicable
NS: Non-significant
OHSS: Ovarian hyperstimulation syndrome
OR: Odds ratio
PCOS: Polycystic ovary syndrome
PN: Pronucleus/pronuclei
SD: Standard deviation
t0: Time of fertilization
t2-t9: Time of cleavage to 2- till 9-cells
tB: Time of full blastocyst stage
TE: Trophectoderm
tEB: Time of expanded blastocyst stage
TESE: Testicular sperm extraction
tPNa: Time of pronuclear appearance
tPNf: Time of pronuclear fading
tSB: Time of start of blastulation
