# Supplementary material for: Human oocyte area is associated with preimplantation embryo usage and early embryo development: the Rotterdam Periconception Cohort
Source: J Assist Reprod Genet. 2023 May 2;40(6):1495–506. doi: 10.1007/s10815-023-02803-1 (PMC10310608; doi:10.1007/s10815-023-02803-1)
Supplement: Supplementary file 1 — Supplementary file1 (DOCX 32 KB) [file 10815_2023_2803_MOESM1_ESM.docx]

**Human oocyte area is associated with preimplantation embryo usage and early development: The Rotterdam Periconception Cohort**

**Supplemental Methods**

*Statistical analysis*

Analyses on the post-implantation clinical treatment outcomes were performed on the transferred embryos. The dichotomous treatment outcomes, like positive pregnancy test, fetal heartbeat (at 12 weeks gestational age) and live birth, were analyzed using logistic regression.

**Table S1.** Baseline characteristics of the included and excluded study population of the VIRTUAL EmbryoScope Study.

|  | **Included**  **study participants**  n= 378^1^ | **Excluded**  **study participants**  n= 75^1^ | p-value |
| --- | --- | --- | --- |
| **Maternal characteristics** |  |  |  |
| Maternal age, years | 33.7±4.9 | 33.9±5.2 | 0.83 |
| Nulliparous | 282 (74.6) | 57 (76.0) | 0.91 |
| Geographic origin |  |  | 0.83 |
| Dutch | 290 (79.0) | 60 (82.2) |  |
| Western | 17 (4.6) | 3 (4.1) |  |
| Non-Western | 60 (16.3) | 10 (13.7) |  |
| Education |  |  | 0.97 |
| Low | 21 (5.7) | 4 (5.5) |  |
| Middle | 146 (39.6) | 30 (41.1) |  |
| High | 202 (54.7) | 39 (53.4) |  |
| Maternal BMI (kg/m2) | 24.7 [21.9, 27.8] | 24.3 [21.5, 27.2] | 0.42 |
| BMI >25 | 175 (46.4) | 33 (44.6) |  |
| BMI >30 | 63 (16.7) | 12 (16.2) |  |
| Mean arterial pressure, *at study entry* | 86.00 [79.33, 93.33] | 86.0 [80.3, 93.7] | 0.95 |
| Folic acid supplement use | 345 (93.5) | 68 (93.2) | 0.89 |
| Multivitamin use | 186 (54.5) | 36 (53.7) | 1.00 |
| Alcohol use | 157 (42.9) | 31 (42.5) | 0.90 |
| Smoking | 50 (13.7) | 12 (16.4) | 0.75 |
| **Treatment factors** |  |  |  |
| Subfertility diagnosis |  |  | 0.10 |
| Only male factor | 170 (45.0) | 32 (42.7) |  |
| Only female factor | 86 (22.8) | 21 (28.0) |  |
| Combined | 80 (21.2) | 10 (13.3) |  |
| Unexplained | 42 (11.1) | 12 (16.0) |  |
| PCOS | 76 (20.1) | 12 (16.0) | 0.51 |
| Fertilization method  IVF  ICSI |  |  | 0.50 |
|  | 124 (32.8) | 17 (22.7) |  |
|  | 254 (67.2) | 27 (61.4) |  |
| ICSI with ejaculated sperm | 145 (38.4) | 13 (17.3) |  |
| ICSI with surgical retrieved sperm | 109 (28.8) | 14 (18.7) |  |
| Ovarian stimulation, GnRH-agonist | 102 (27.2) | 5 (6.7) | 0.19 |
| No. of embryos transferred |  |  | <0.001* |
| No ET | 18 (4.8) | 17 (39.5) |  |
| Single ET | 312 (82.5) | 23 (53.5) |  |
| Double ET | 48 (12.7) | 3 (7.0) |  |
| Days of culture |  |  | 0.162 |
| 3 | 252 (66.7) | 38 (71.7) |  |
| 5 | 117 (30.9) | 14 (18.6) |  |
| Culture media |  |  | 0.81 |
| SAGE 1-Step | 329 (87.0) | 68 (90.7) |  |
| GTL Vitrolife | 49 (13.0) | 7 (9.3) |  |
| **Treatment outcomes** |  |  |  |
| Oocytes aspirated | 8.00 [5, 13] | 9 [4, 12] | 0.34 |
| Total fertilized oocytes | 5 [3, 7] | 3 [1, 6] | 0.002* |
| Fertilization rate | 0.80 [0.63, 1.00] | 0.71 [0.44, 0.89] | 0.05 |
| Total usable embryos | 3 [2.00, 5] | 2 [1, 3] | 0.14 |
| Embryo usage rate | 0.67 [0.50, 0.86] | 0.54 [0.38, 1.00] | 0.71 |
| OHSS | 26 (6.9) | 1 (1.3) | <0.001* |
| hCG + | 147 (43.2) | 7 (26.9) | 0.12 |
| GS + | 133 (60.9) | 6 (23.1) | 0.13 |
| Fetal heart beat, + | 120 (64.7) | 3 (11.5) | 0.02* |
| Live birth(s) | 111 (34.9) | 2 (7.7) | 0.02* |
| Cumulative pregnancy | 178 (50.8) | 18 (24.0) | 0.008* |

Data is presented as mean ± standard deviation, median [interquartile range] or number of individuals (percentage). *Significant differences between included and excluded participants are reported with a p<0.05. ^1^Missing data rates by variable were reported and excluded from the total sum of participants or the denominator during analyses. Abbreviations: BMI, body mass index; ET, embryo transfer; GnRH, gonadotropin releasing hormone; GS, gestational sacs; hCG, human chorionic gonadotropin; ICSI, intracytoplasmic sperm injection; IVF, in vitro fertilization; PCOS, polycystic ovary syndrome.

**Table S2.** Oolemma area of the discarded fertilized and unfertilized oocytes after ICSI.

| **Discarded ICSI oocytes** n=1174 | | | | |
| --- | --- | --- | --- | --- |
|  | | **Oocytes with PN**  n=604 | **Oocytes without PN**  n=527 | p-value |
| Oolemma area (µm²) | t0 | 10376.50 [9939.00, 10831.00] | 10563.00 [10043.00, 11265.00] | <0.001* |
|  | tPNa | 9684.50 [9278.00, 10137.75] | n/a | n/a |
|  | tPNf | 9679.00 [9230.50, 10144.50] | n/a | n/a |
| Time (h) | tPNa | 8.20 [7.00, 10.10] | n/a | n/a |
|  | tPNf | 24.60 [22.30, 27.70] | n/a | n/a |
| Shrinking rate (µm²/hour) | | -24.17 [-38.84, -13.42] | n/a | n/a |

Data is presented as median [interquartile range]. tPNf was not applicable for IVF oocytes without PN so no data could be shown. *Significant differences with a p<0.05. Abbreviations: ICSI, intracytoplasmic sperm injection; PN, pronuclei; t0, time of fertilization; tPNa, time of pronuclear appearance; tPNf, time of pronuclear fading.

**Table S3.** Oolemma area of the discarded fertilized oocytes based on the number of pronuclei stratified by fertilization method.

| **Discarded ICSI fertilized oocytes** n=604 | | | | |
| --- | --- | --- | --- | --- |
|  |  | **Oocytes with 2PN**  n=528 | **Oocytes with <2PN or >2PN**  n=76 | p-value |
| Oolemma area (µm²) | t0 | 10355.00 [9932.00, 10830.00] | 10407.00 [10067.50, 10937.00] | 0.213 |
|  | tPNa | 9733.00 [9283.00, 10146.00] | 9603.00 [9210.00, 9938.25] | 0.055 |
|  | tPNf | 9680.00 [9233.00, 10146.00] | 9633.00 [9135.00, 9987.00] | 0.448 |
| Time (h) | tPNa | 8.10 [6.90, 10.00] | 9.10 [7.50, 11.30] | 0.050 |
|  | tPNf | 24.60 [22.39, 27.50] | 24.80 [22.10, 29.36] | 0.629 |
| Shrinking rate (µm²/hour) | | -23.36 [-38.18, -12.98] | -28.72 [-42.85, -17.80] | 0.187 |
| **Discarded IVF fertilized oocytes** n=277 | | | | |
|  |  | **Oocytes with 2PN**  n=270 | **Oocytes with <2PN or >2PN**  n=7 | p-value |
| Oolemma area (µm²) | tPNf | 9735.00 [9221.25, 10141.00] | 9327.00 [8935.50, 9601.50] | 0.169 |
| Time (h) | tPNf | 27.26 [24.34, 30.70] | 28.80 [26.55, 30.75] | 0.533 |

Data is presented as median [interquartile range]. *Significant differences with a p<0.05. Abbreviations: ICSI, intracytoplasmic sperm injection; IVF, in vitro fertilization; PN, pronuclei; t0, time of fertilization; tPNa, time of pronuclear appearance; tPNf, time of pronuclear fading.

**Table S4**. Logistic regression model of the association between oocyte area at tPNf in transferred ICSI oocytes (n=242, range 7483 to 11930 µm²) and post-implantation clinical outcomes.

|  |  |  | **Oocyte area (10^-3^** **µm²)** | | | | | |
| --- | --- | --- | --- | --- | --- | --- | --- | --- |
| **Clinical outcomes** | **Case number** | **Crude** | | | | **Adjusted** | | |
|  |  | Estimate | | OR (95% CI) | p-value | Estimate | OR (95% CI) | p-value |
| hCG+ | 107 | 0.091 | | 1.095 (0.725, 1.660) | 0.666 | 0.289 | 1.336 (0.862, 2.084) | 0.196 |
| GS+ | 98 | 0.090 | | 1.094 (0.722, 1.667) | 0.671 | 0.262 | 1.300 (0.840, 2.026) | 0.241 |
| FHB+ | 88 | 0.035 | | 1.036 (0.678, 1.589) | 0.870 | 0.160 | 1.174 (0.755, 1.833) | 0.477 |
| LB+ | 83 | 0.076 | | 1.079 (0.702, 1.667) | 0.729 | 0.202 | 1.224 (0.784, 1.922) | 0.375 |

Model adjusted for maternal age, ovarian stimulation and fertilization method, sperm retrieval method and culture medium. Only single embryo transfers are included in this analysis. *Significant differences with a p<0.05. Abbreviations: CI, confidence intervals; ICSI, intracytoplasmic sperm injection; OR, odds ratio; FHB, fetal heart beat; GS, gestational sac(s); hCG, human chorionic gonadotrophin; LB, live birth.
